# Single Event Resolution of Plant Plasma Membrane Protein Endocytosis by TIRF Microscopy

**DOI:** 10.3389/fpls.2017.00612

**Published:** 2017-04-24

**Authors:** Alexander Johnson, Grégory Vert

**Affiliations:** Institute for Integrative Biology of the Cell (I2BC), CNRS/CEA/Univ. Paris-Sud, Université Paris-SaclayGif-sur-Yvette, France

**Keywords:** imaging techniques, endocytosis, TIRF microscopy, Arabidopsis, plants, trafficking

## Abstract

Endocytosis is a key process in the internalization of extracellular materials and plasma membrane proteins, such as receptors and transporters, thereby controlling many aspects of cell signaling and cellular homeostasis. Endocytosis in plants has an essential role not only for basic cellular functions but also for growth and development, nutrient delivery, toxin avoidance, and pathogen defense. The precise mechanisms of endocytosis in plants remain quite elusive. The lack of direct visualization and examination of single events of endocytosis has greatly hampered our ability to precisely monitor the cell surface lifetime and the recruitment profile of proteins driving endocytosis or endocytosed cargos in plants. Here, we discuss the necessity to systematically implement total internal reflection fluorescence microcopy (TIRF) in the Plant Cell Biology community and present reliable protocols for high spatial and temporal imaging of endocytosis in plants using clathrin-mediated endocytosis as a test case, since it represents the major route for internalization of cell-surface proteins in plants. We developed a robust method to directly visualize cell surface proteins using TIRF microscopy combined to a high throughput, automated and unbiased analysis pipeline to determine the temporal recruitment profile of proteins to single sites of endocytosis, using the departure of clathrin as a physiological reference for scission. Using this ‘departure assay’, we assessed the recruitment of two different AP-2 subunits, alpha and mu, to the sites of endocytosis and found that AP2A1 was recruited in concert with clathrin, while AP2M was not. This validated approach therefore offers a powerful solution to better characterize the plant endocytic machinery and the dynamics of one’s favorite cargo protein.

## Introduction

Endocytosis is the process of transporting cell surface or extracellular materials, including proteins, lipids and nutrients, into the cell *via* invaginations of small vesicles that pinch off from the plasma membrane. While there are many different forms of endocytosis, the most characterized is clathrin-mediated endocytosis (CME). It is defined by a coat of clathrin which forms around the invaginating vesicle. At least 60 key endocytosis accessory proteins (EAPs) are conserved between mammalian and yeast CME (Merrifield and Kaksonen, 2014). Some of these key EAPs also have homologs in plants, suggesting that plant CME might use the same mechanisms as these other systems (Baisa et al., 2013).

CME is best understood in mammalian and yeast systems (McMahon and Boucrot, 2011; Lu et al., 2016) where over 60 conserved key EAPs have been characterized and for which details of the physiological role and precise temporal dynamics are defined (McMahon and Boucrot, 2011; Merrifield and Kaksonen, 2014; Lu et al., 2016). These findings have shown that CME can be broken down into five distinct steps; nucleation, cargo selection, coat assembly, scission, and uncoating. Each stage requires a different subset of EAPs to facilitate the overall propagation of creating a vesicle from the plasma membrane.

In the past few years, a number of plant plasma membrane proteins have been shown to utilize the CME pathway. These include the some of the PIN auxin efflux carrier and the PIP2 aquaporin (Dhonukshe et al., 2007), subunits of the cellulose synthase (Bashline et al., 2013), the IRT1 root iron transporter (Barberon et al., 2014), and the plant steroid hormone receptor BRI1 (Di Rubbo et al., 2013; Martins et al., 2015), among others. In contrast to mammalian and yeast systems, little is known about CME in plants and at present, there are only a handful of reports which really begin to address the precise molecular mechanisms of plant CME. Identification of plant EAP homologs of mammalian or yeast EAPs (Chen et al., 2011; Baisa et al., 2013) has led to genetic manipulations of CME to demonstrate that some of the EAP homologs are indeed key proteins for plant CME. For example, use of clathrin heavy chain mutants interfered with the recycling and polarization of PIN proteins, thus demonstrating that CME is indeed important in cell surface processes (Kitakura et al., 2011). All the canonical Adaptor Protein 2 (AP-2) subunits are also conserved in plants (Happel et al., 2004; Chen et al., 2011; Yamaoka et al., 2013), and also appear to be important for the internalization of some cell surface reporters and normal plant development (Bashline et al., 2013; Di Rubbo et al., 2013; Fan et al., 2013; Kim et al., 2013). However, there are some key proteins which appear to have not been conserved between plants and other model systems (Gadeyne et al., 2014; Zhang et al., 2015), suggesting that CME in plants is partly reliant upon unique plant EAPs, and therefore potentially utilizes different mechanisms of CME.

**FIGURE 1:**
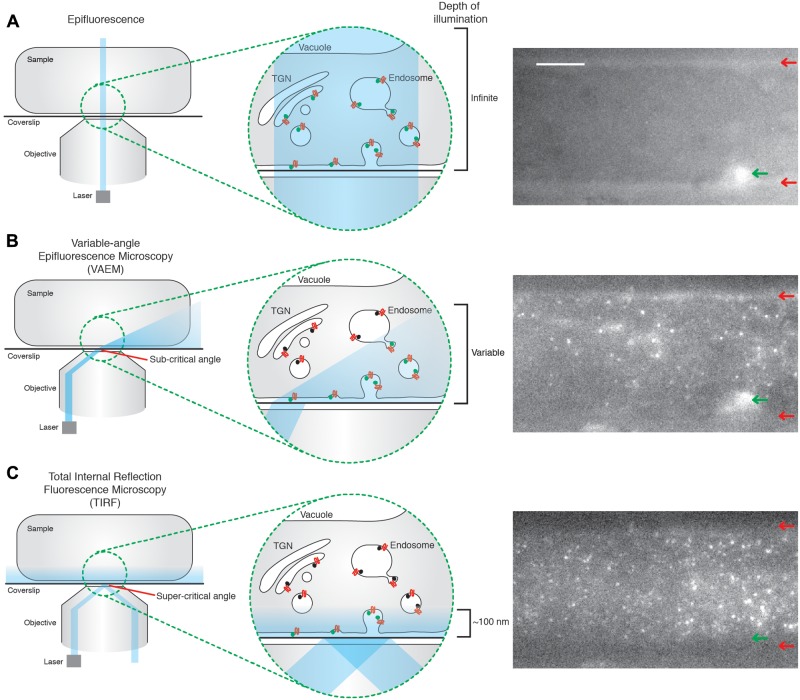
**Total internal reflection fluorescence microcopy (TIRF) produces uniform illumination of the cell surface.** **(A)** Epifluorescence; the illumination beam is perpendicular to the coverslip and passes directly through the sample. This excites fluorophores throughout the sample, producing an image in which it is impossible to determine the z position of the excited fluorophore, as shown in the example image. The edges of the cell are clearly visible (red arrows), as well as a structure within the cell (green arrow). **(B)** Variable-angle epifluorescence microscopy (VAEM); the illumination beam hits the interface of two media with different refractive indexes (for example, the glass coverslip and the sample), at a sub-critical angle. The beam is refracted toward the coverslip, which produces a ‘cone’ shape of illumination, where the further from the point of refraction the greater the depth of penetration. This results in a non-uniform illumination of the cell surface, as evidenced in the example image where one of the edges from the cell is visible (red arrows), as well as a structure within the cell body (green arrow). **(C)** TIRF; the illumination beam hits the interface of two media with different refractive indexes (for example, the glass coverslip and the sample), at the critical angle. The majority of the beam is reflected, but a low energy evanescent wave is generated which can penetrate the sample up to ∼100 nm. The energy of this wave is directly proportional to the distance from the point of total reflection, thus illuminating fluorophores only on, or very close to, the cell surface in a uniform manner; as seen in the example image. The edges of the cell (red arrows), and the structure within the cell body (green arrow) visible in the other methods of imaging are no longer visible under TIRF illumination. The example images are of the same Arabidopsis root tip, expressing TPLATE-GFP, but with the angle of the illumination beam altered for each circumstance. Scale bar: 500 μm.

One of the main reasons mammalian and yeast systems enjoy such detailed physiological characterization of CME compared to plants is the use of specific technologies which allow direct examination of single events of CME *in vivo*. Much of the current characterization of plant CME has relied heavily upon indirect, and often static, biochemical and pharmacological approaches using confocal microscopy that fail to capture the often subtle roles of EAPs at CME sites with any temporal resolution (Aguet et al., 2013). Single event resolution is, however, critical for defining precise physiological roles for the EAPs, as it directly tracks and allows quantification of the dynamics of EAPs at the site of their physiological action. One of the key technologies facilitating single event imaging of CME is total internal reflection fluorescent microscopy (TIRF). This method of imaging makes use of a low energy evanescent wave which is generated when the illumination laser undergoes total reflection at the interface of two different media of different reflective indexes; for example, between a coverslip and imaging medium (Axelrod, 2001). The energy of the evanescent wave is directly proportional to the distance away from the point of reflection, typically penetrating samples up to 100 nm. TIRF therefore only excites fluorophores on, or very close to the cell surface (Axelrod, 2001). This property makes of TIRF a great method to image cell surface processes such as CME. This is in contrast to traditional epifluorescence approaches, where the illumination source passes directly through the whole sample, thereby stimulating fluorophores present on structures deeper within the cell and making it impossible to determine if the fluorescent signal originates from the cell surface (**Figure 1A**). To overcome the drawbacks of epifluorescence, spinning disk confocal microscopy has often been used in the plant field to look at cell surface processes. Use of the pinholes allows one to select the cell surface plane and facilitates rapid acquisition. However, due to the nature of the pinholes, a lot of photons are discarded and the z-resolution is still limited to ∼500 nm. This makes spinning disk microscopy unsuitable for tracking weak signals and focusing with great precision on cell surface EAPs aggregating at sites of CME (Mettlen and Danuser, 2014).

Another approach to image the plant cell surface is the variable angle evanescent microscopy (VAEM), also called ‘pseudo-TIRF’. VAEM relies on refraction, rather than reflection as in TIRF, of the illumination laser at the interface of two different media of different reflective indexes (Konopka et al., 2008). The illumination wave is refracted to almost parallel angles to the coverslip, thus exciting fluorophores near the coverslip. However, the depth of penetration of the refracted wave is not uniform across the sample (**Figure 1B**). The further away from the point of reflection, the broader the illumination beam is, thus allowing deeper stimulation of fluorophores within the cell (Wan et al., 2011). This non-uniform illumination allows one to distinguish between TIRF and VAEM while imaging (**Figures 1B,C**). TIRF is therefore more sensitive, produces evenly illuminated images with a higher signal to noise ratio allowing the tracking of weaker signals, and is more accurate to quantify cell surface lifetime due to uniform z-resolution. As such, TIRF is now seen as the standard method for CME investigation in both mammalian and yeast systems, despite yeast having a cell wall (Rappoport, 2008; Loerke et al., 2009). Encouragingly, a handful of studies showed that TIRF is now possible in plants (Vizcay-Barrena et al., 2011; Wan et al., 2011).

Along with specific cell surface imaging techniques, automated image analysis algorithms have been developed, which allowed high throughput, unbiased and rapid analysis of CME TIRF images (Jaqaman et al., 2008; Aguet et al., 2013; Langhans and Meckel, 2014). Thousands of fluorescent signals can be automatically detected, tracked and quantified from a time-lapse movie of the cell surface in under an hour. This has allowed the analysis of huge datasets and thus accurately depicted CME with a high power of statistical significance (Loerke et al., 2009). Although a few recent studies in plants have used such algorithms (Wang et al., 2015), the vast majority rely on manual analysis methods using ImageJ (Konopka et al., 2008; Bashline et al., 2013; Martins et al., 2015). This mostly involves making kymographs of a time-lapse TIRF or VAEM movie and manually measuring each line of fluorescence in ImageJ. This is a very time-consuming endeavor, and can take months to produce small datasets. Furthermore, this technique is not as sensitive at detecting particles as a computer algorithm, and is open to selection bias toward the brighter or persisting signals. Manual tracking is also unsuitable for proteins which are dense on the cell surface, as there is too much noise to accurately measure single traces of proteins.

Taking together that TIRF is indeed possible in plants and that plant images are suitable for automated particle detection programs, we developed a reliable method for directly studying single events of CME in plants with high temporal and spatial resolution. We established a protocol for allowing TIRF imaging to be performed on the Arabidopsis root tip and successfully applied single endocytic event detection and tracking scripts to the images to allow rapid, high throughput and unbiased calculation of the cell lifetime of key EAPs. Finally, we detailed the development of a dual channel physiological departure assay, which allows the fine characterization of EAPs at single sites of CME. Altogether, this provides the plant endocytosis community with powerful imaging pipelines to greatly increase our understanding of plant CME, thus bridging the gap in knowledge between other model systems.

## Materials and Methods

### Plant Material

Plants expressing AP2A1-GFP (35S::AP2A1-GFP) were gifted from Dr. Russinova (VIB, Belgium). CLC-tagRFP (pRPS5A::CLC2-tagRFP) and TPLATE-GFP (pLat52::TPLATE-GFP) were gifted from Dr. Van Damme (VIB, Belgium). AP2M-YFP (pAP2M::AP2M-YFP) was gifted by Dr. Gu (Pennsylvania State University, USA). CLC-GFP (pCLC::CLC2-GFP) was provided by Dr. Lin (Chinese Academy of Sciences, Beijing, China). DRP1c-GFP (pDRP1c::DRP1c-GFP) was gifted by Dr. Bednareck (University of Wisconsin, USA).

For dual color analysis, plants expressing the EAPs fused to GFP were crossed with the pollen from CLC-tagRFP plants, and the F1 progenies were used.

### Growth Conditions

Plants were plated on half-strength Linsmaier and Skoog medium (½ LS) and incubated in the dark at 4°C for 2–4 days. Plates were transferred to a heated light chamber (21°C with cycles of 16 h light/8 h dark) and incubated for 10 days prior to imaging.

### TIRF Imaging and Analysis

**FIGURE 2:**
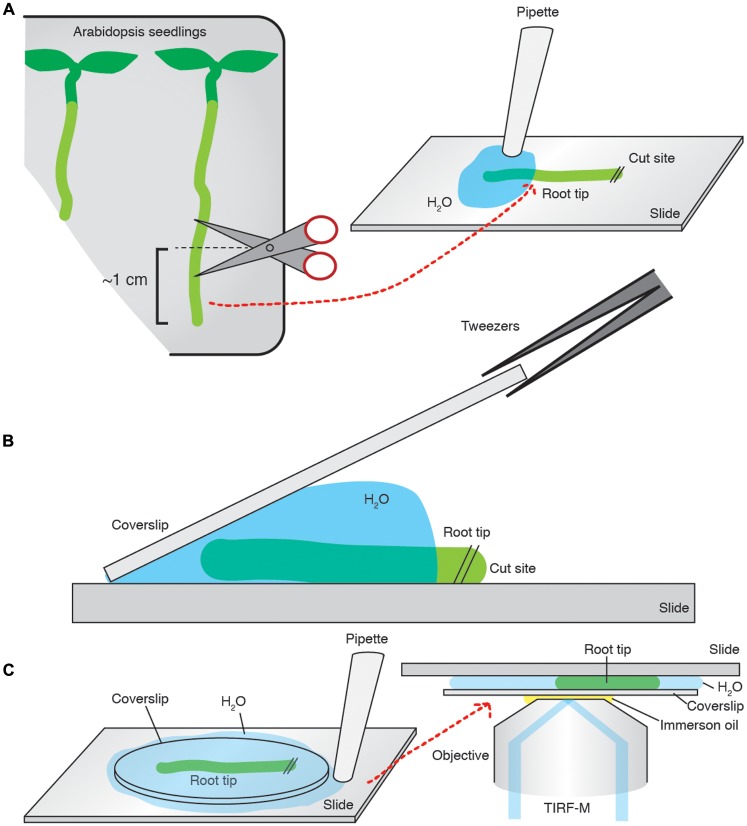
**Arabidopsis root tip preparation for TIRF imaging.** **(A)** ∼1 cm of the root is cut and transferred onto a slide and bathed in imaging medium. **(B)** A pre-cleaned coverslip is placed on the sample, and slowly laid flat. **(C)** The excess medium is then aspirated away. The slide is mounted onto the microscope and imaged.

Total internal reflection fluorescent microscopy samples were prepared in the following manner. Coverslips (Borosilicate glass, thickness 1, from VWR) were cleaned with a 0.01% (w/v) Decon 90, 100 mM NaOH solution and then sequentially washed with ddH_2_0, 100% ethanol and then acetone. The root of interest was cut to ∼1 cm above the root tip, and transferred to a microscope slide where it was bathed in an excess of imaging medium before a pre-cleaned coverslip was placed on top of the sample. The excess medium was then aspirated to create a compression of the coverslip onto the sample, ensuring that the epidermal cells were in direct contact with the coverslip and that the root was immobilized without damage. The slide was subsequently mounted onto a Nikon Eclipse Ti microscope equipped with a Nikon APO TIRF 100/1.49 oil immersion objective (**Figure 2**). The excitation wavelengths used were 491 nm and, or, 561 nm for GFP and tagRFP, respectively, provided by a 100-mW diode laser Toptica AOTF. An emission filter Chroma ET 405/491/561/642 was used in conjunction with a Coolsnap HQ2 camera (photometrics).

For single channel analyses, images were acquired at 1 s intervals, for a duration of 5 min. The time-lapse movies were subjected to only the particle detection and tracking algorithms from the cmeAnalysis package (Aguet et al., 2013) using Matlab 2013b (Mathworks). Using customized homemade Matlab scripts (available upon request), the complete raw trajectories were screened so that only tracks which had both a duration greater than 5 s and that lasted less than the total length of the movie, and tracks which were not present in the first of last five frames of the movie were included in the cell surface lifetime calculation. The data from multiple experiments were combined to produce cell surface lifetime values.

Dual channel time-lapse movies were acquired sequentially at 500 ms intervals, at an interval of 1 s per channel, for a duration of 5 min. Each channel was subjected only to the particle detection and tracking stages of the cmeAnalysis package in Matlab 2013b (Mathworks), as described above. Custom homemade Matlab scripts (available upon request) were used to examine if the raw cmeAnalysis trajectories of each channel overlapped. If a 1 frame overlap was found, the different channel trajectories were paired. Paired trajectories were subjected to the departure analysis if the appearance and disappearance of the secondary channel trajectory of each channel were within 5 s of the reference channel trajectory’s appearance and disappearance (Aguet et al., 2013). The mean lifetime value of these paired trajectories was calculated, and paired trajectories where the reference trajectory lifetime equaled this value (±1 s) were combined and aligned to the moment of the disappearance of the reference channel, thus creating a departure trace. The mean departure traces from multiple experiments were combined, normalized and plotted to produce a robust recruitment profile of the EAP of interest to the moment of the departure of the reference signal.

## Results and Discussion

### Establishing TIRF, Automated Particle Detection, and Tracking Analysis in Arabidopsis Roots

The use of TIRF in plants is relatively new and there have been only a few reports precisely detailing how it is achieved. We therefore set out to establish and share a reliable and reproducible method for TIRF imaging and analyzing TIRF data in the Arabidopsis root. As the evanescent wave only penetrates depths up to 100 nm (Axelrod, 2001), a major requirements of TIRF is mounting the sample so it is in direct contact with the coverslip. We developed a simple method for successfully mounting Arabidopsis roots onto cover slips, which ensures contact of the epidermal cells with the coverslip and immobilization without damaging the sample (**Figure 2**). While imaging an intact system provides analysis of CME in its physiological context, it also presents difficulty in selecting the same cell type and developmental stage. To overcome this, we use the lateral root cap as a reference point. The first suitable epidermal cells generating TIRF images, usually located 5–13 cells up from the end of the lateral root cap, were selected for analyses.

**FIGURE 3:**
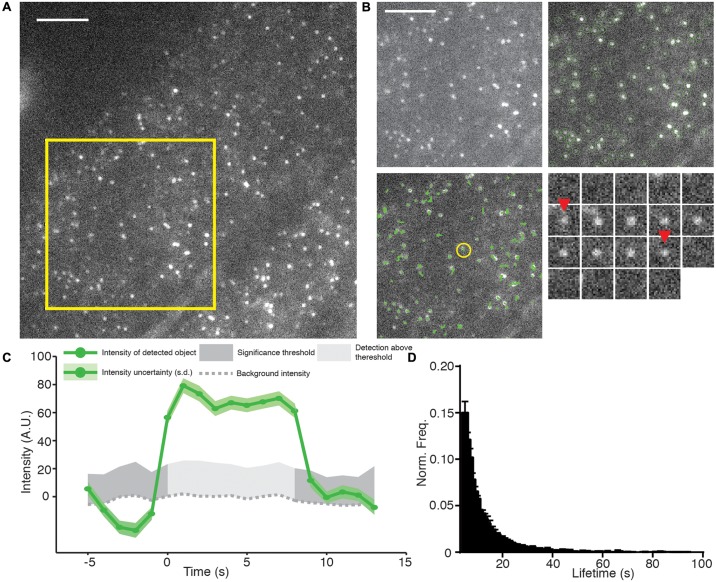
**Example of TIRF, automated detection, particle tracking, and lifetime calculation of AP2A1-GFP.** **(A)** An example TIRF image of Arabidopsis root tips expressing AP2A1-GFP. **(B)** Top left panel; magnified view of the yellow square in **(A)** shows that AP2A1 forms district foci with a high signal to noise ratio under TIRF illumination. Top right panel; Each AP-2 foci is detected by the detection algorithm (green circles). Bottom left panel; each detected particle can be tracked over the duration of its presence on the plasma membrane (green lines). Bottom right; shows a time series (1 s intervals) of a single particle (yellow circle in the bottom left panel) where red arrows denoted appearance and disappearance of the spot. **(C)** Intensity trace of the time series in **(C)**. Only when the intensity is above the uncertainty level of the local background (dark gray) is it regarded as valid time point in the particles trajectory. **(D)** Histogram of the lifetimes of AP2A1-GFP on the cell surface (*n* = 3; 14,203 tracks). Scale bars: 500 μm.

Plants expressing a GFP labeled alpha subunit of the canonical CME AP-2 adaptor protein (AP2A1-GFP) were subjected to TIRF using these methods (**Figure 3A**). AP2A1-GFP forms discrete foci on the cell surface, as expected, and these spots have a high signal to noise ratio. Importantly, the image has a uniform intensity across the whole sample, indicating that TIRF illumination was used. Once a suitable cell is selected, a time-lapse movie is acquired with a time interval between frames of 1 s for a duration of 5 min. The movie is then subjected to automated particle detection and tracking. To do this, we used only the detection and tracking parts of the cmeAnalysis package (Aguet et al., 2013). The cmeAnalysis package was selected for use as it outperforms other detection and tracking programs available, like uTrack and Imaris (Mettlen and Danuser, 2014). It uses a model fitting mode of detection, which allows the use of statistical test to determine if an object fits the expected point spread function of the specific fluorophore used, and if the fluorescent intensity is statistically significant compared to the local background (Aguet et al., 2013). cmeAnalysis detected AP2A1-GFP spots with a very high degree of accuracy (**Figure 3B**).

The cmeAnalysis package can complete tracks which includes gaps in them (Aguet et al., 2013). This is critical to determine accurate lifetimes when weak signals can fluctuate close to the limit of detection (Jaqaman et al., 2008). For example, at the early stages of CME, only a small numbers of proteins are present before they polymerize and produce a brighter stable signal. Our observations indicate that the spots detected in our experiments can be tracked over the length of the movie (**Figure 3B**). Once the tracking is complete, the fluorescence intensity and lifetime of every particle are examined (**Figure 3C**). To do this, we extracted the raw tracking output of the cmeAnalysis program and used custom made Matlab scripts to screen the trajectories. Tracks that started too close to the start, or to the end, of the movie were excluded to ensure complete recording. Furthermore and as previously described, tracks less than 5 s were excluded to prevent a bias of short tracks caused during the gap linking stage (Aguet et al., 2013). The lifetimes from multiple experiments were combined to produce a robust mean value (**Figure 3D**). Using the combination of the cmeAnalysis algorithm, and our own scripts, for AP2A1-GFP, we calculated a cell surface lifetime of 12.74 s from three different roots and 14,203 individual total tracks. There is very little variation between the different roots imaged (results from experimental replicates were within a range of 1.43 s of each other), therefore demonstrating that our imaging and analysis methods are robust.

**FIGURE 4:**
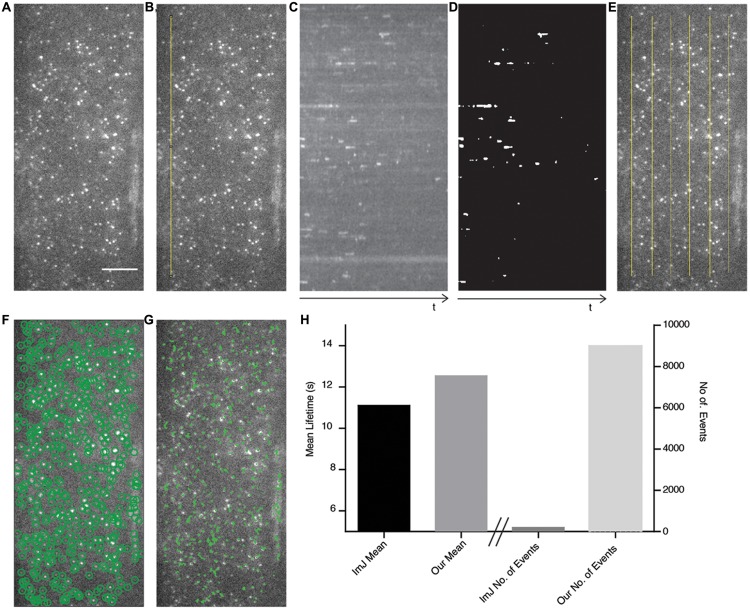
**Comparison of ImageJ-based analysis method with our automated method.** **(A)** The AP2A1-GFP TIRF image is used as a test case to compare both methods. Scale bar: 500 μm. **(B)** A linear region of interest (ROI) (yellow line) is drawn onto the image stack. **(C)** A kymograph of the ROI in **(B)** is produced and a Gaussian blur filter is applied. **(D)** The kymograph is segmented and the particles are measured using ImageJ. **(E)** Six different kymographs were produced from the image and combined. **(F)** Our method automatically detects (green circles) all the particles present in the image. **(G)** The detected particles are tracked over their appearance in the movie (green lines). **(H)** The mean lifetimes of AP2A1 and number of particles included in the analysis of the ImageJ (ImJ) method and the method presented in this paper.

To compare our method with a recently published semi-automated analysis method (Higaki, 2015), which is reliant upon ImageJ and human input, we examined a cell expressing AP2A1-GFP using TIRF illumination (**Figure 4A**) and compared the cell surface lifetime of AP2A1, the number of tracks analyzed, and the time taken to generate results. For the ImageJ analysis, a linear region of interest (ROI) is drawn on the stack image (**Figure 4B**). This ROI is then resliced to produce a kymograph, and a Gaussian blur filter applied (**Figure 4C**). The kymograph is then segmented and the tracks of the particles measured for their length using ImageJ (**Figure 4D**), as described by Higaki (2015). The results are then copied in to Excel spreadsheet for further processing. This process was repeated six times for the single cell (**Figure 4E**). Our analysis method successfully automatically detected almost all the particles present in the image and was able to track them over their appearance and disappearance within the movie (**Figures 4F,G**), requiring human input only for information about the specific imaging setup used. Overall, the cell surface lifetime results were within 1.44 s of each other between the two methods (11.10 and 12.54 s for the ImageJ-based and our own method, respectively) (**Figure 4H**). There were however striking differences in the number of tracks measured (**Figure 4H**). Finally, the ImageJ-based method involving a lot more human interaction with the analysis took significantly longer than our method to detect only a limited number of events.

**FIGURE 5:**
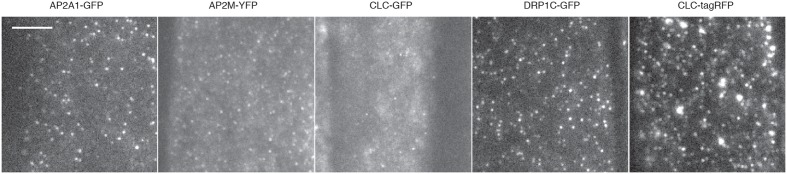
**Examples of TIRF images and cell surface lifetimes of EAPs.** Example TIRF images of Arabidopsis root tips expressing AP2A1-GFP, AP2M-YFP, CLC-GFP, DRP1c-GFP, and CLC-tagRFP. Scale bar: 500 μm.

To further validate our method, we examined the cell surface lifetime of DRP1c-GFP, a potential EAP which has been previously reported to have a cell surface lifetime of 17.7 s in developing root tip cells (Konopka et al., 2008). DRP1c-GFP formed discrete foci on the cell surface under TIRF illumination (**Figure 5**), agreeing with previous attempts to image it on the cell surface (Konopka et al., 2008). After detection and tracking, we decided to use the same filtering criteria of the lifetime values as previously used (Konopka et al., 2008), and found a lifetime of 16.03 s from 53,574 tracks analyzed (**Table 1**). Further to this, we also examined the lifetimes of clathrin light chain (CLC) tagged with GFP or tagRFP. Both GFP and tagRFP tagged CLC formed discrete foci on the cell surface (**Figure 5**), and showed no significant difference in their lifetimes (*p* = 0.113, unpaired *t*-test; four independent experiments with 16,824 CLC-GFP events and 12,449 CLC-tagRFP events) (**Table 2**). Therefore, the choice of fluorophore or promoter does not appear to bias cell surface lifetimes in our specific case.

**Table 1:** The cell surface lifetime data of DRP1c-GFP was filtered as described by Konopka et al. (2008).

Protein	Tag	Lifetime (s)	±*SE*	#Events	#Independent roots
DRP1C	GFP	16.0281	0.1007	53,574	6


*Lifetimes are presented in seconds, and SE represents the standard error to the mean.*

**Table 2:** The cell surface lifetime values of studied EAPs.

Protein	Tag	Lifetime (s)	±*SE*	#Events	#Independent roots
AP2A1	GFP	12.553	0.084946	14,203	3
AP2M	YFP	15.87	0.04928	96,228	5
CLC	GFP	10.439	0.067309	16,824	4
DRP1C	GFP	20.305	0.11702	31,420	6
CLC	Tag-RFP	11.671	0.092766	12,449	4


*Lifetimes are presented in seconds, and SE represents the standard error to the mean.*

Altogether, this indicated that we successfully established a robust and powerful pipeline to allow TIRF, automated detection, tracking and rapid lifetime calculation of cell surface proteins in root epidermal cells.

### Development of a Dual Channel Clathrin Departure Assay in Plants

**FIGURE 6:**
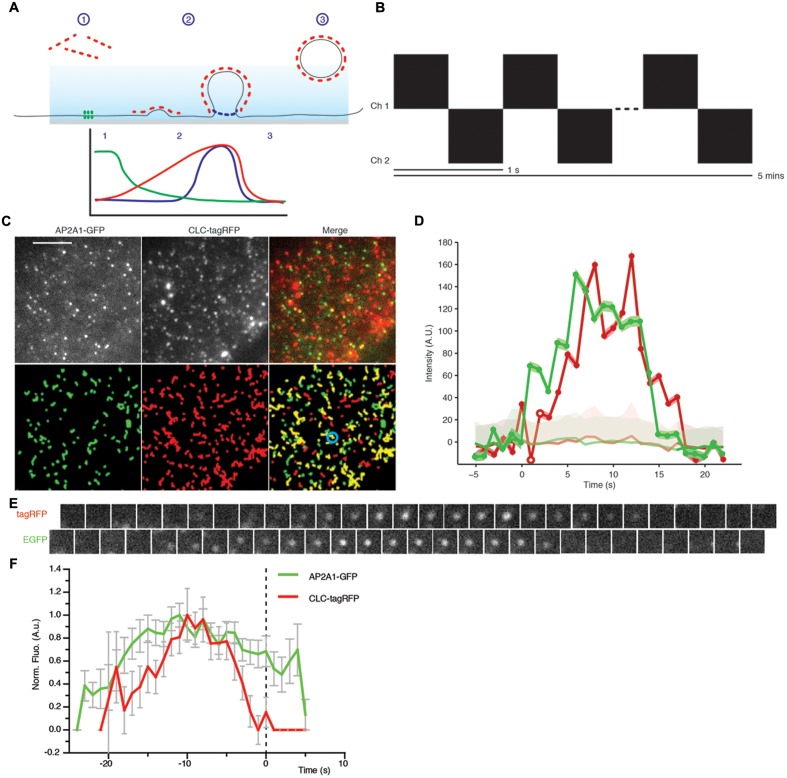
**Principle of the departure assay.** **(A)** Schematic showing the departure assay. The evanescent wave (blue) penetrates the sample ∼100 nm, thus only exciting fluorophore on the plasma membrane. (1) Before clathrin (red dots) is recruited to sites of endocytosis, it is outside the evanescent wave, therefore the fluorophore is not excited. (2) As the vesicle invagination develops, more clathrin is recruited resulting in an increase of fluorescent intensity. (3) Once the clathrin-coated vesicle is scissioned away from the plasma membrane, it moves away and thus out of the evanescent wave excitation, resulting in a decrease of fluorescent intensity. Dual channels allow another protein to be examined in conjunction with the clathrin trace. Once aligned to the moment of departure of the clathrin trace, we can determine the dynamics of recruitment to single sites of CME. For example, the green trace depicts what one would expect for a protein which is present before the vesicle invagination, and the blue trace represents a protein which is required later in the propagation of CME. **(B)** Schematic showing how images were acquired. Each channel was imaged sequentially with a time interval of 1 s per image for a duration of 5 min. **(C)** Upper panel; example TIRF images of Arabidopsis root tips expressing AP2A1-GFP and CLC-tagRFP. Lower panel; trajectories of detection particles in the upper panel for each channel. CLC tracks which have an overlapping green track are shown in yellow. **(D)** Example fluorescent intensity plots of a single trajectory (blue circle in **C**), the trajectories are only significant when they overcome the uncertainly level of the local background (faded red/green). **(E)** Example time series of the trajectories of the highlighted spot (blue circle in **C**). **(F)** Combined mean departure plots of AP2A1-GFP and CLC-tag RFP (*n* = 3, 300 departure events). Scale bar: 500 μm.

The physiological significance of single channel cell surface lifetimes is hard to interpret, as it lacks a meaningful physiological context. Without a reference in the experiment to compare the cell surface lifetime to, it is impossible to determine the role of the protein at the cell surface. We therefore developed a dual channel TIRF imaging system using CLC as a reference for CME, where scission of the clathrin-coated vesicle from the membrane is visualized by the sharp decrease of CLC fluorescence (**Figure 6A**) (Merrifield et al., 2002; Mattheyses et al., 2011). Tracks from the EAP and CLC can be combined from multiple single events of CME and aligned to the moment the CME vesicle departs from the membrane, thus allowing one to define with great precision when EAPs are recruited to single events of CME (**Figure 6A**).

As a test case for dual TIRF imaging-based departure assay, we monitored the recruitment of AP-2 compared to CLC. Plants expressing AP2A1-GFP were crossed with CLC-tagRFP-expressing plants and the F1 progeny was subjected to this departure assay. Time series movies were captured in both the red and green channels sequentially, with a frequency of 1 s between frames (**Figure 6B**). Both AP2A1 and CLC formed discrete foci on the cell surface, as expected (**Figure 6C**). Many more CLC spots were observed at the cell surface compared to AP2A1, consistent with the existence of another adaptor complex in plant that may serve different pools of CME (Gadeyne et al., 2014). Particles in both channels were detected and tracked (**Figure 6C**), as described above. The raw tracking data was extracted from the cmeAnalysis package, and we used homemade Matlab scripts to examine the overlap between CLC and AP2A1 trajectories. Trajectories with an overlap in space and time of 1 frame were paired. From this population of paired trajectories, we then selected trajectories where the CLC lifetime was equal to the mean (±1 s) of all the CLC trajectories. Paired trajectories where the green track started, or ended more than five frames before or after the red track were discarded, to ensure that only overlapping of trajectories corresponding to a given CME event were analyzed and avoid coincidental overlapping tracks, as previously described (Aguet et al., 2013). The GFP overlapping ‘partner’ track was then aligned to the moment of the CLC departure (**Figures 6D,E**). Every departure traces from multiple experiments were normalized and combined to generate a recruitment profile of AP2A1 to sites of CME marked by CLC departure (**Figure 6F**). The CLC-tagRFP fluorescence trace is in accordance with the classical model of CME with CLC-tagRFP gradually polymerizing at the cell surface while the vesicle invaginates (indicated by the increase of fluorescence), and then rapidly decreasing as the vesicle moves away from the cell surface (indicated by the decrease in fluorescence). Consistent with the AP-2 being an adaptor for CME driving the recruitment of clathrin to the cell surface (Shih et al., 1995; McMahon and Boucrot, 2011), it arrived at the sites of CME before CLC (**Figure 6F**). Further to this, AP2A1 shares a similar departure trace as CLC, thus indicating that the AP2A1 subunit is involved in plant CME.

### Differential Recruitment of AP-2 Subunits to Sites of CME

AP-2 is the canonical adaptor protein for CME in mammalian systems (McMahon and Boucrot, 2011). AP-2 is a complex made of different subunits, and each subunit appears to play a key role in the propagation of CME. For example, the alpha subunit has cargo recognition sites (i.e., DxF, FxDxF, and WVxF motifs) (Benmerah et al., 1996; Brett et al., 2002; Jha et al., 2004; Walther et al., 2004), and is involved in membrane binding and recruitment of other EAPs (Chen et al., 1998). The beta subunit binds clathrin (Shih et al., 1995), and cargo via the di-leucine motif (Hofmann et al., 1999). The mu subunit binds cargo which contain the YxxΦ (Φ being a bulky hydrophobic residue) motif (Ohno et al., 1995). Many of the subunits are conserved in plants, and overall AP-2 appears to be important for internalization of proteins and development (Chen et al., 2011; Bashline et al., 2013; Di Rubbo et al., 2013; Fan et al., 2013; Kim et al., 2013; Yamaoka et al., 2013). However, AP-2 is not the major adaptor in plants, as deletion still allows production of a viable plant (Bashline et al., 2013; Kim et al., 2013; Yamaoka et al., 2013; Gadeyne et al., 2014). Recent evidence in both plants and *Caenorhabditis elegans* suggests that the AP-2 complex functions as two distinct hemi-complexes composed as alpha/sigma and beta/mu, which have differing partially independent roles (Gu et al., 2013; Wang et al., 2016). Notably, the mu subunit would mediate the recruitment of the alpha subunit to the cell surface (Wang et al., 2016). Consistently, the lifetimes of the alpha and mu subunits at the cell surface were found to be significantly different (*p* = 0.0335, unpaired *t*-test; AP2A1, *n* = 3 independent experiments with 14,203 events; AP2M, *n* = 4 independent experiments with 96,228 events) (**Figure 5**).

**FIGURE 7:**
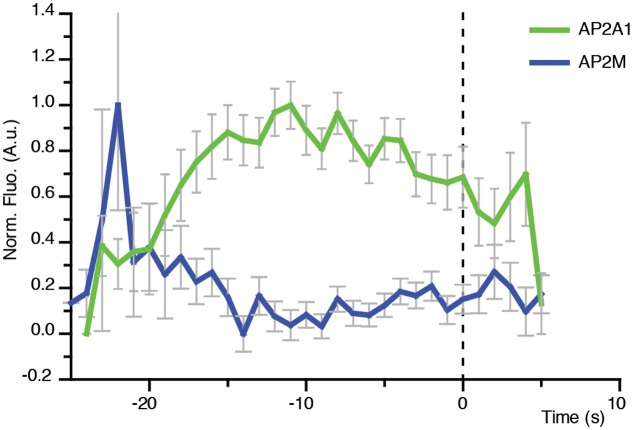
**Direct visualization of AP-2 alpha and mu subunits to single sites of CME.** Combined mean departure plots of AP2A1-GFP (green) (*n* = 3 cells, 300 departure events) and AP2M-YFP (blue) (*n* = 5 cells, 619 departure events).

In contrast to the concomitant recruitment of AP2A1 with CLC (**Figure 6F**), the recruitment profile of the AP-2 mu subunit at the cell surface only showed a spike prior to the arrival of CLC (**Figure 7**). This suggests that the AP-2 mu subunit is not required for the propagation of the CME event *per se*, but might in fact be required during the early stages of CME. This agrees with pharmacological and genetic manipulations which showed that loss of the AP-2 mu subunit adversely affected the recruitment of the other AP-2 subunits to the cell surface (Wang et al., 2016).

## Conclusion

In contrast to routine analysis of cell surface internalization processes performed by traditional confocal and spinning disk microscopy, the utilization of TIRF allows the live and direct examination of the cell surface free from noise, the precise lifetime and departure profile calculation. While TIRF is the imaging approach of choice when examining cell surface processes, there are some limitations. For example, as TIRF is reliant upon the evanescent wave, which typically can only penetrate samples up to 100 nm, the cell of interest must lie flat and in direct contact with the coverslip in order to be illumined. This restricts the imaging volume of TIRF in intact organisms to only the outside cell layer. Therefore, TIRF is only applicable in plants to certain cell types such as epidermal cells or pollen. It also relies on genetic manipulation of the sample to label the protein of interest with a fluorophore. This could alter the structural properties of how proteins would assemble and interact, further strengthening the necessity to validate the functionality of the fusion protein. While the tagging of clathrin and AP-2 has routinely been done in many systems and shown not to interfere with their function (Rappoport and Simon, 2008), this has to be established for any new protein imaged. The fluorophore used or the expression level of the tagged proteins imaged however do not seem to influence the cell surface lifetime, at least for clathrin.

Overall, the ability to make precise temporal studies of EAPs is instrumental to reconstitute the sequential events of EAP recruitment and to better characterize endocytosis in plants. With a large body of plant research currently focusing on the dynamics and endocytosis of cell surface receptors and transporters (Luu and Maurel, 2013; Luschnig and Vert, 2014; Zelazny and Vert, 2014; Ben Khaled et al., 2015), TIRF and semi-automated image analysis provide suitable solutions to unravel the molecular mechanisms driving CME, and one’s favorite cargo.

## Author Contributions

AJ and GV: designed the experimental strategy and analyzed data; wrote the manuscript.

## Conflict of Interest Statement

The authors declare that the research was conducted in the absence of any commercial or financial relationships that could be construed as a potential conflict of interest.
